# Comprehensive Profiling Analysis of CD209 in Malignancies Reveals the Therapeutic Implication for Tumor Patients Infected With SARS-CoV-2

**DOI:** 10.3389/fgene.2022.883234

**Published:** 2022-06-17

**Authors:** Jinyuan Li, Shuzhao Chen, Yang Li, Ziang Zhu, Hanying Huang, Weida Wang, Yao Yang, Yang Liang, Lingling Shu

**Affiliations:** ^1^ State Key Laboratory of Oncology in South China, Collaborative Innovation Center for Cancer Medicine, Sun Yat-sen University Cancer Center, Guangzhou, China; ^2^ Department of Hematologic Oncology, Sun Yat-sen University Cancer Center, Guangzhou, China; ^3^ Institute of Pharmaceutics, School of Pharmaceutical Sciences, Sun Yat-sen University, Shenzhen, China; ^4^ State Key Laboratory of Pharmaceutical Biotechnology, The University of Hong Kong, Hong Kong, SAR, China

**Keywords:** CD209, malignancies, immune infiltration, SARS-CoV-2, bioinformatics

## Abstract

Coronavirus disease 2019 (COVID-19), which is known to be caused by the virus severe acute respiratory syndrome coronavirus-2 (SARS-CoV-2), is characterized by pneumonia, cytokine storms, and lymphopenia. Patients with malignant tumors may be particularly vulnerable to SARS-CoV-2 infection and possibly more susceptible to severe complications due to immunosuppression. Recent studies have found that CD209 (DC-SIGN) might be a potential binding receptor for SARS-CoV-2 in addition to the well-known receptor ACE2. However, pan-cancer studies of CD209 remain unclear. In this study, we first comprehensively investigated the expression profiles of CD209 in malignancies in both pan-carcinomas and healthy tissues based on bioinformatic techniques. The CD209 expression declined dramatically in various cancer types infected by SARS-CoV-2. Remarkably, CD209 was linked with diverse immune checkpoint genes and infiltrating immune cells. These findings indicate that the elevation of CD209 among specific cancer patients may delineate a mechanism accounting for a higher vulnerability to infection by SARS-CoV-2, as well as giving rise to cytokine storms. Taken together, CD209 plays critical roles in both immunology and metabolism in various cancer types. Pharmacological inhibition of CD209 antigen (D-mannose), together with other anti-SARS-CoV-2 strategies, might provide beneficial therapeutic effects in specific cancer patients.

## Introduction

COVID-19, a recently emerging respiratory viral illness, has become a worldwide pandemic, with more than 343 million infection cases and more than 5.5 million deaths as of 21 January 2022 (Organization, 2021). Since malignant tumors and related tumor treatments could seriously impair the immune function, patients with malignancies are especially susceptible to SARS-CoV-2 infection (Liang et al., 2020). In addition, hyperinflammation and a subsequent “cytokine storm,” which is characterized by the increased expression levels of different proinflammatory cytokines (e.g., TNF and IL-6), have been observed in severe COVID-19 cases (Song et al., 2022), which indicates that immune regulation plays an essential role in the pathogenesis of COVID-19 (Nicholls et al., 2003; Mahallawi et al., 2018).

Recent studies have found that SARS-CoV-2 has a close relationship with SARS-CoV, which share approximately 80% homology. Both of these viruses utilize angiotensin-converting enzyme-2 (ACE2) as their receptors to infect and enter host cells (Zhou et al., 2020). In addition, studies have suggested that specific tumor patients and smokers are more susceptible to infection by SARS-CoV-2 due to the higher expression of ACE2 (Dai et al., 2020; van Zyl-Smit et al., 2020). In addition to ACE2, CD209 (DC-SIGN) has been found to play a crucial role in the pathogenicity of SARS-CoV (Yang et al., 2004; Balbi et al., 2021; Wang et al., 2021). CD209, which is capable of capturing pathogens, is mainly expressed on dendritic cells and plays an important role in antigen transportation, which is particularly exemplified in HIV-1 (Geijtenbeek et al., 2000; Simmons et al., 2003; Geurtsen et al., 2010; Alam et al., 2021). Studies have demonstrated that CD209 can bind directly in a glycan-dependent manner with SARS-CoV-2’s S glycoprotein and cooperates with ACE2 for the viral entry of SARS-CoV, which is mediated through endocytosis (Yang et al., 2004; Han et al., 2007; Xu et al., 2020). Recent studies also showed that one of the COVID-19 risk variants, rs657152-A, was associated with increases in the CD209 antigen and facilitated the infection of SARS-CoV-2, SARS-CoV, and other viruses (Jeffers et al., 2004; Katz et al., 2020).

Alternative clinical therapeutics were a combination of supportive patient care and several antiviral drugs, including treatments with antimalarials (hydroxychloroquine) and nucleotide analogs (remdesivir) (Rome and Avorn, 2020; Consortium et al., 2021). However, hydroxychloroquine and remdesivir were later proven to be ineffective (Consortium et al., 2021). Despite Paxlovid having been approved as the special antiviral drug for COVID-19 (Mahase, 2021; Extance, 2022), the development of treating SARS-CoV-2 infections is still challengeable at present. Treatment for SARS-CoV-2 infections according to immunopathology is increasingly focused on the healthy population (Li et al., 2020). A better understanding of the relative protease action of CD209 and the effects of receptor binding would be helpful for predicting latent additional routes for virus infection and developing novel antiviral immunotherapy.

Here, we demonstrated a profiling study on CD209 expression levels in both pan-cancer tissues and healthy tissues and the association of CD209 with infiltrating immune cells in tumor patients. We probed the underlying therapeutic implications of CD209 in SARS-CoV-2 infections in carcinoma patients. These findings might provide important clues for specific carcinoma patients to prevent infection with SARS-CoV-2 and alleviate the cytokine storm that occurs in infected patients. Given that CD209 antigen (D-mannose) has been revealed as an effective therapy for congenital disorders of glycosylation type I (de Lonlay and Seta, 2009; Schneider et al., 2011), it could suppress autoimmune diabetes and inflammation in mouse airways effectively by promoting Treg cell generation and activating the TGF-β pathway (Zhang et al., 2017). This study will shed light on the therapeutic potency of CD209 antigens and help prevent or attenuate SARS-CoV-2 infection in specific tumor patients.

## Methods

### Gene Expression Analysis

The expression profiles of CD209 mRNA and protein in different tissues, immune cell types, and cancer types were obtained from the online database, namely, The Human Protein Atlas website (https://www.proteinatlas.org). The expression data for CD209 mRNA and protein in pan-cancer can be downloaded from the UALCAN analysis website (http://ualcan.path.uab.edu/analysis.html). We also analyzed the single-cell RNA-seq data to search for CD209 expression data in the nasopharynx immune cells of COVID-19 patients, which is available on the UCSC cell browser portal (http://www.cells.ucsc.edu/).

### Survival Analysis

The Kaplan–Meier plotter (Győrffy, 2021) was used to obtain the overall survival (OS) significance map data of CD209 in all cancer types (https://kmplot.com/analysis/), and the “log-rank test” module was used to test the hypothesis.

### Immuno-Infiltration Analysis

We used the default “Immune module” available in the TIMER2 database (http://timer.cistrome.org/) to analyze the relationship of CD209 expression with the abundances of NK cells, CD4^+^ T cells, CD8^+^ T cells, B cells, neutrophils, macrophages, cancer-associated fibroblasts, and dendritic cells by their corresponding gene modules. In a chosen cancer type, the correlation module could generate user-defined gene expression scatter plots, as well as their relative statistical significance by Spearman’s correlation and corresponding estimation.

### Expression of CD209 in Different Cell Lines and Organs Infected With SARS-CoV/SARS-CoV-2

To study the CD209 expression in SARS-CoV-2- or SARS-CoV-infected cells/organs, we reanalyzed the public available transcriptomic data (GSE52920, GSE171110, and GSE161881) from the Gene Expression Omnibus database (GEO), together with a proteome dataset (IPX0002393000) from the iProX database (http://111.198.139.98/page/home.html) (Nie et al., 2021). In particular, GSE52920 contained six lung tissues of mice, including three infected SARS-CoV and three controls. Vero E6 cells were mock-infected or infected with SARS-CoV-2 at an moi of 2 in the GSE161881 dataset. GSE171110 contained the whole-blood gene expression profiles of 44 COVID-19 patients and 10 healthy donors. RNA-seq data from tests between COVID-19 patients and their healthy controls were obtained from the IPX0002393000 dataset. A graphical visualization of CD209 data was prepared using Graph Prism 7.0 software (GraphPad Prism Software, United States).

### Expression of CD209 in Smokers With LUAD and LUSC

Three datasets including GSE10072, GSE68465, and GSE50081 were employed to investigate the expression of CD209 in LUAD or LUSC patients with different smoking histories. All patients with smoking histories were divided into three groups: never smoked, former smoker, and current smoker. Graphs were prepared using Graph Prism 7.0 software.

### Statistical Analysis

We evaluated the gene correlations by Spearman’s correlation. Their correlation strength was determined using the absolute value guidelines as follows: 0.80–1.0 as “very strong,” 0.60–0.79 as “strong,” 0.40–0.59 as “medium,” 0.20–0.39 as “weak,” and 0.00–0.19 as “very weak.” Statistical significance was assessed by *p* values < 0.05.

## Results

### CD209 Expression in Healthy Human Tissue

**FIGURE 1:**
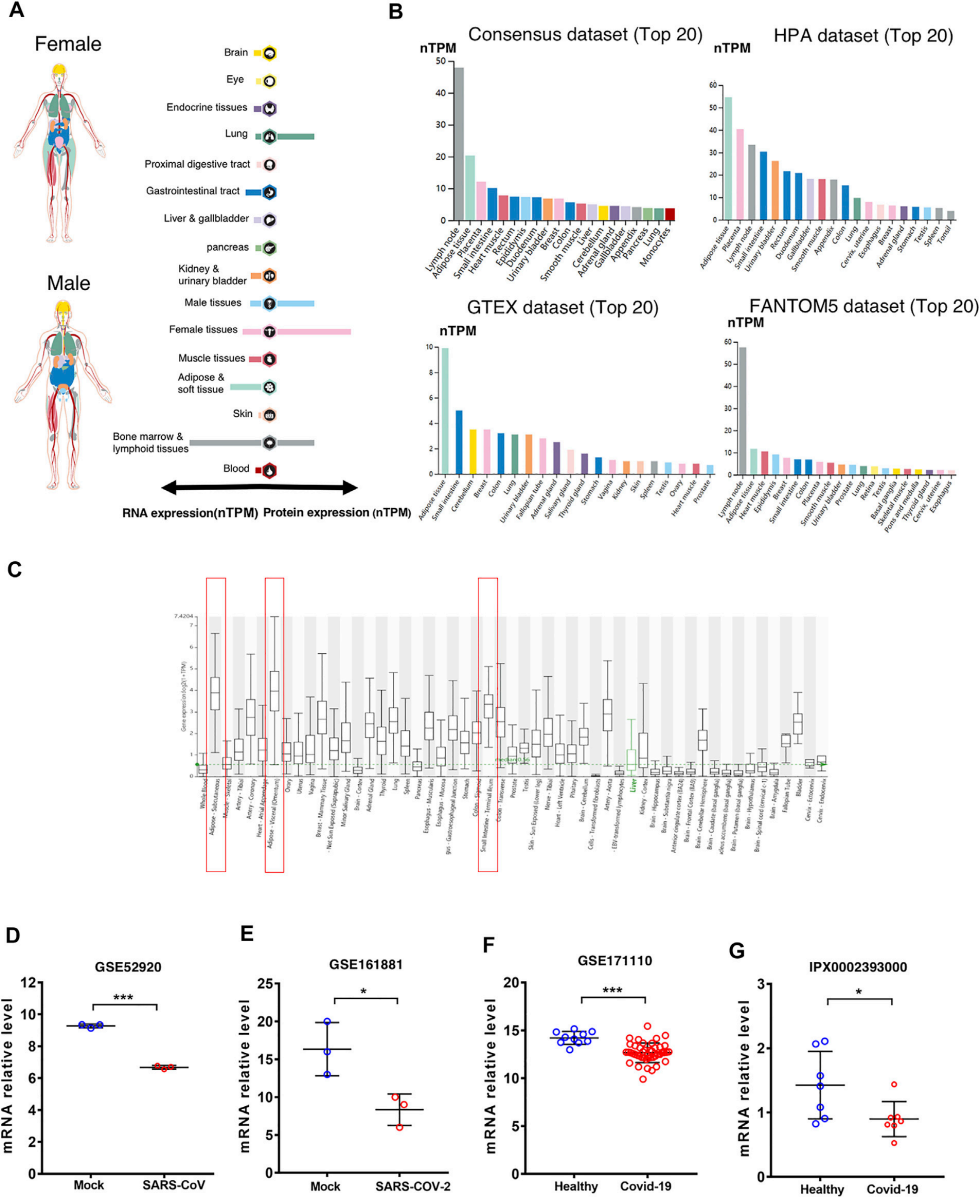
Expression of CD209 in human tissues and organs. **(A)** mRNA and protein expression profiles of CD209 in all tissues and organs of healthy people analyzed in the Tissue Atlas database; RNA and protein expression summary shows the normalized expression (nTPM) values. Color-coding is based on tissue groups. **(B)** Top 20 tissues or organs with CD209 mRNA expression in four databases (Consensus dataset, Fantom5 dataset, GTEx dataset, and HPA dataset). Four database’s data are reported as nTPM (normalized protein-coding transcripts per million). Color-coding is based on tissue groups. **(C)** CD209 mRNA expression in various tissues was analyzed in the HCCDB database. **(D)** mRNA level of CD209 in lung of mice infected with SARS-CoV (*n* = 6). **(E)** mRNA expression level of CD209 in vero E6 cells line transfected mock, SARS-CoV-2 (moi = 2) for 8 h (*n* = 6). **(F)** CD209 levels in peripheral blood of COVId-19 patients (*n* = 44) and their healthy controls (*n* = 10). **(G)** CD209 expression in testis of COVID-19 patients (*n* = 7) and their healthy controls (*n* = 7). Data are represented as mean ± SD. *p < 0.05, ***p < 0.01, ***p < 0.001(Student’s t-test). Abbreviation: moi, multiplicity of infection; TPM, transcripts per million.

The CD209 gene was most abundantly expressed in the lymph nodes, adipose tissue, and muscle tissues, while the CD209 protein was distributed mostly in the lymph nodes and lungs in male and female tissues (Figure 1A). Ultimately, we analyzed the CD209 gene expression in four public datasets (HPA, Consensus, GTEx, and FANTOM5). The expression of CD209 was detected at the highest levels within the adipose tissue and lymph nodes and at relatively lower levels in the small intestine, placenta, heart muscle, and urinary bladder. Intriguingly, adipose tissue demonstrated the abundant expression of CD209 in the GTEx and HPA public datasets, which was in line with the current concept that obese patients are more susceptible to infection by SARS-CoV-2 (Figure 1B). Recent studies have shown that SARS-COV-2 can directly infect primary DCs and macrophages, which may lead to the destruction of human LNs and spleens and subsequent lymphocytopenia (Feng et al., 2020). However, ACE2 was barely expressed in lymphoid cells. Our data showed that CD209 was a highly expressed gene in macrophages and DCs. Moreover, we investigated the CD209 gene expression in a variety of tissues through the HCCDB database. We found that adipose tissue and the small intestine had the highest CD209 gene expression (Figure 1C), which was consistent with the expression of ACE2. In summary, CD209 was expressed abundantly in various healthy human tissues, especially in adipose tissue, small intestine, and lymph nodes, which had higher susceptibility to infection by SARS-CoV-2.

To identify the CD209 expression in SARS-CoV-2-infected versus infected organs/cell lines, we reanalyzed the public available transcriptomic datasets (GSE52920, GSE161881, GSE171110, and iProX IPX0002393000) that have been uploaded recently to GEO and iProX. Compared to the healthy controls, the mRNA abundance of CD209 demonstrated significant decreases in the lungs of mice (Figure 1D, Supplementary Table S1), Vero E6 cells (Figure 1E, Supplementary Table S2) and peri-blood (Figure 1F, Supplementary Table S3), and testes of patients (Figure 1G, Supplementary Table S4) infected with SARS-CoV/SARS-CoV-2. Together with previous discoveries, these data strongly suggested that CD209 might play a critical role in mediating SARS-CoV-2 infection in patients.

### Landscape Profiling of CD209 in Pan-Carcinoma

**FIGURE 2:**
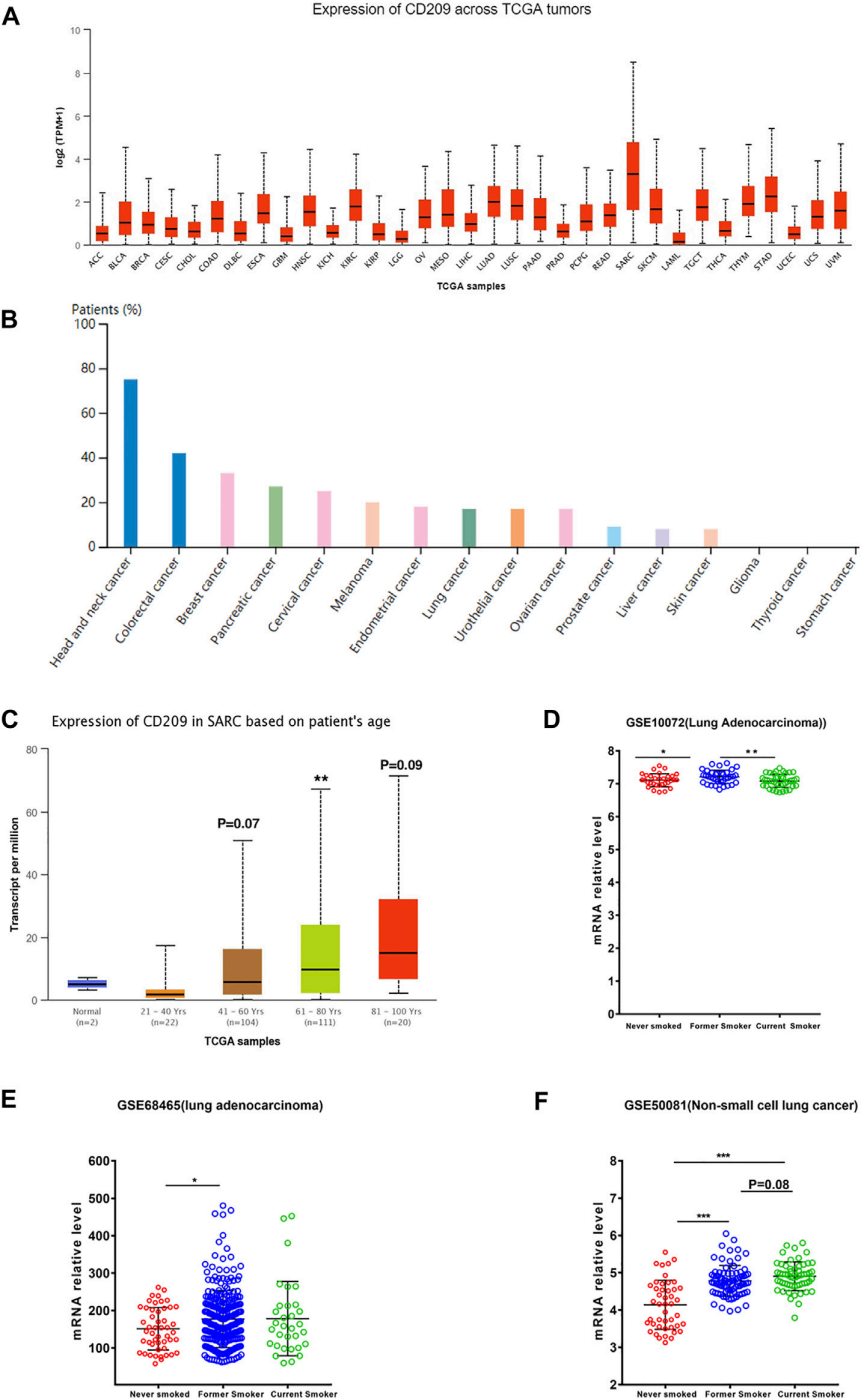
Expression profile of CD209 in pan-cancers. **(A)** RNA expression level of CD209 in pan-cancers in the TCGA dataset. **(B)** Protein expression level of CD209 in pan-cancers in CAB045970 dataset. **(C)** Expression of CD209 increased in SARC patients associated with patient’s age. **(D)** Expression of CD209 was upregulated in former smokers with LUAD in dataset GSE10072 (*n* = 58). **(E)** Expression of CD209 was upregulated in former smokers with LUAD in dataset GSE68465 (*n* = 322). **(F)** Expression of CD209 was upregulated in former smokers and current smokers with LUSC in dataset GSE50081 (*n* = 160). Data are represented as mean ± SD. *p < 0.05, ***p < 0.01, ***p < 0.001(Student’s t-test). Abbreviation: SARC, sarcoma.

Cancer patients suffer a poor prognosis after being infected with SARS-CoV-2 due to their impaired immunity (Li et al., 2020). Thus, we estimated the landscape profile of CD209, a potential SARS-CoV-2 receptor, in pan-carcinoma patients. In the TCGA dataset, we found that CD209 was expressed in various cancer types, while its mRNA abundance was highest in sarcoma (SARC) (Figure 2A), whereas the protein level of CD209 was most enriched in head and neck cancer (Figure 2B). Recent studies demonstrated that elderly patients with sarcoma may be at an increased risk of developing severe complications following SARS-CoV-2 infection (Wagner et al., 2021a; Wagner et al., 2021b). We further showed that the CD209 expression was positively associated with advanced age in SARC patients, especially in those elderly patients over 60 years old (Figure 2C).

Previous studies showed that ACE2 and CD209 were overexpressed in the healthy lungs of smokers, especially former smokers (Cai et al., 2020a; Cai et al., 2020b), but the relevance of this phenomenon in lung cancer remains unclear. Thus, we first examined the expression profiles of former smokers, nonsmokers, and current smokers in LUSC (lung squamous cell carcinoma) and LUAD (lung adenocarcinoma) patients. We found that the CD209 expression was significantly upregulated in former smokers with LUAD (Figures 2D,E, Supplementary Table S5, S6) and current smokers with LUSC (Figure 2F, Supplementary Table S7). These findings might help to interpret why lung cancer patients are more liable to be infected with COVID-19 (Liang et al., 2020).

**FIGURE 3:**
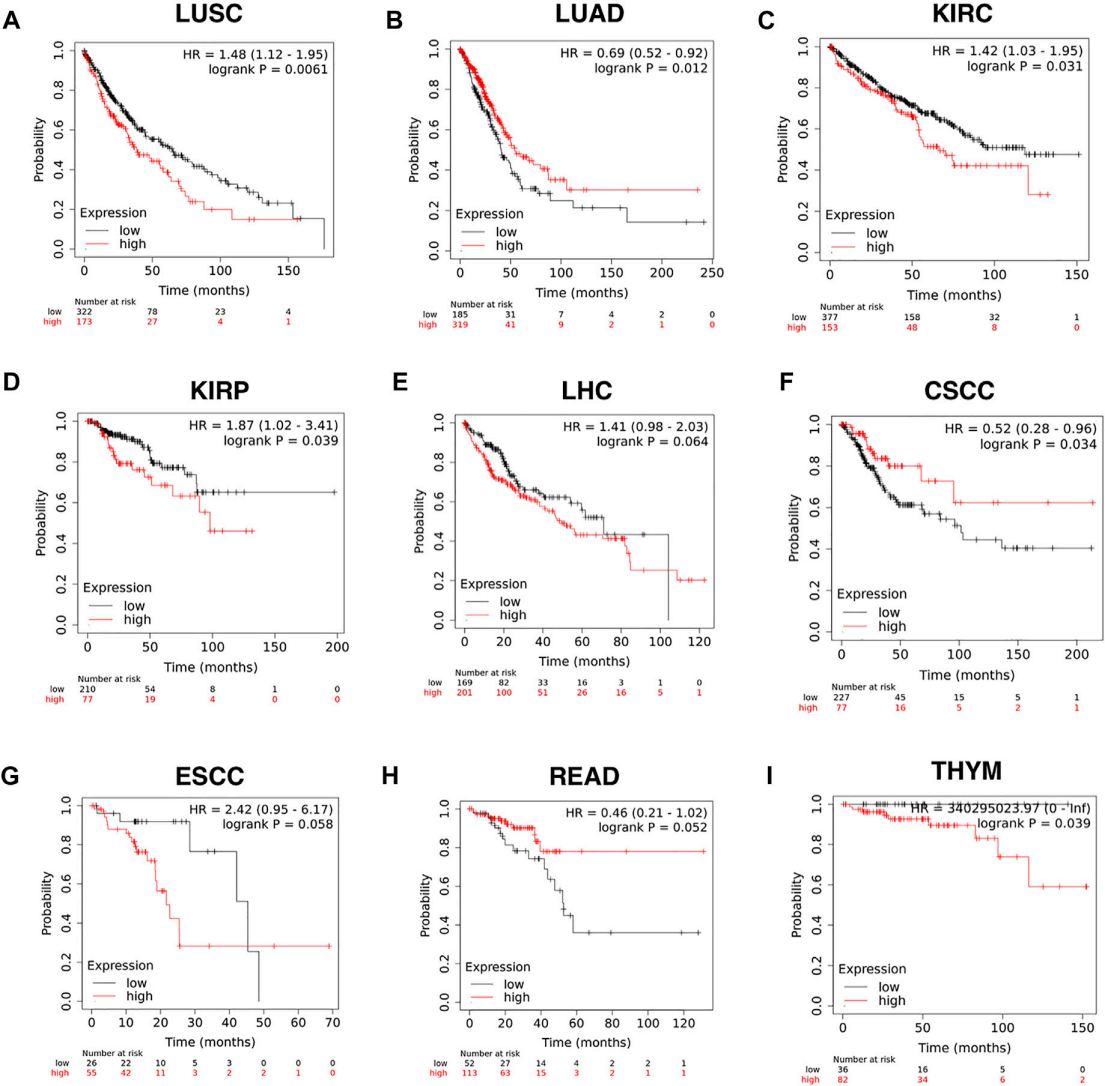
Efficiency of CD209 expression in the survival of specific cancer patients analyzed with the Kaplan–Meier plotter. **(A–I)** Kaplan–Meier plotter of **(A)** LUSC, **(B)** LUAD, **(C)** KIRC, **(D)** KIRP, **(E)** LHC,**(F)** CSCC, **(G)** ESCC, **(H)** READ, and **(I)** THYM based on the expression level of CD209. Abbreviation: LUSC, lung squamous cell carcinoma; LUAD, lung adenocarcinoma; KIRC, kidney renal clear cell carcinoma; KIRP, kidney renal papillary cell carcinoma; CSCC, cervical squamous cell carcinoma; LHC, liver hepatocellular carcinoma; ESCC, esophageal squamous cell carcinoma; READ, rectum adenocarcinoma; THYM, thymoma.

The efficiency of CD209 in the survival of pan-cancer patients was further explored with the Kaplan–Meier plotter (Győrffy, 2021). We found that the reduced CD209 expression was associated with a poor overall survival rate in LUAD (*p* = 0.012, HR = 0.69), READ (rectum adenocarcinoma) (*p* = 0.052, HR = 0.46), and (cervical squamous cell carcinoma) (*p* = 0.034, HR = 0.52). Conversely, the high expression of CD209 predicted poor overall survival in KIRP (kidney renal papillary cell carcinoma) (*p* = 0.039, HR = 1.87), LUSC (*p* = 0.0061, HR = 1.48), KIRC (kidney renal clear cell carcinoma) (*p* = 0.031, HR = 1.42), ESCC (esophageal squamous cell carcinoma) (*p* = 0.058, HR = 2.42), LHC (liver hepatocellular carcinoma) (*p* = 0.064, HR = 1.41), and THYM (thymoma) (*p* = 0.039, HR = 340295023.97) (Figure 3). In summary, these data suggested that the particular distribution of CD209, an alternative receptor of SARS-CoV-2 (Amraei et al., 2021), in different cell types might lead to different prognoses in specific carcinoma patients, indicating that during the COVID-19 epidemic, intensive health care is needed for these cancer patients.

### CD209 Is Highly Expressed in Dendritic Cells and Monocytes

**FIGURE 4:**
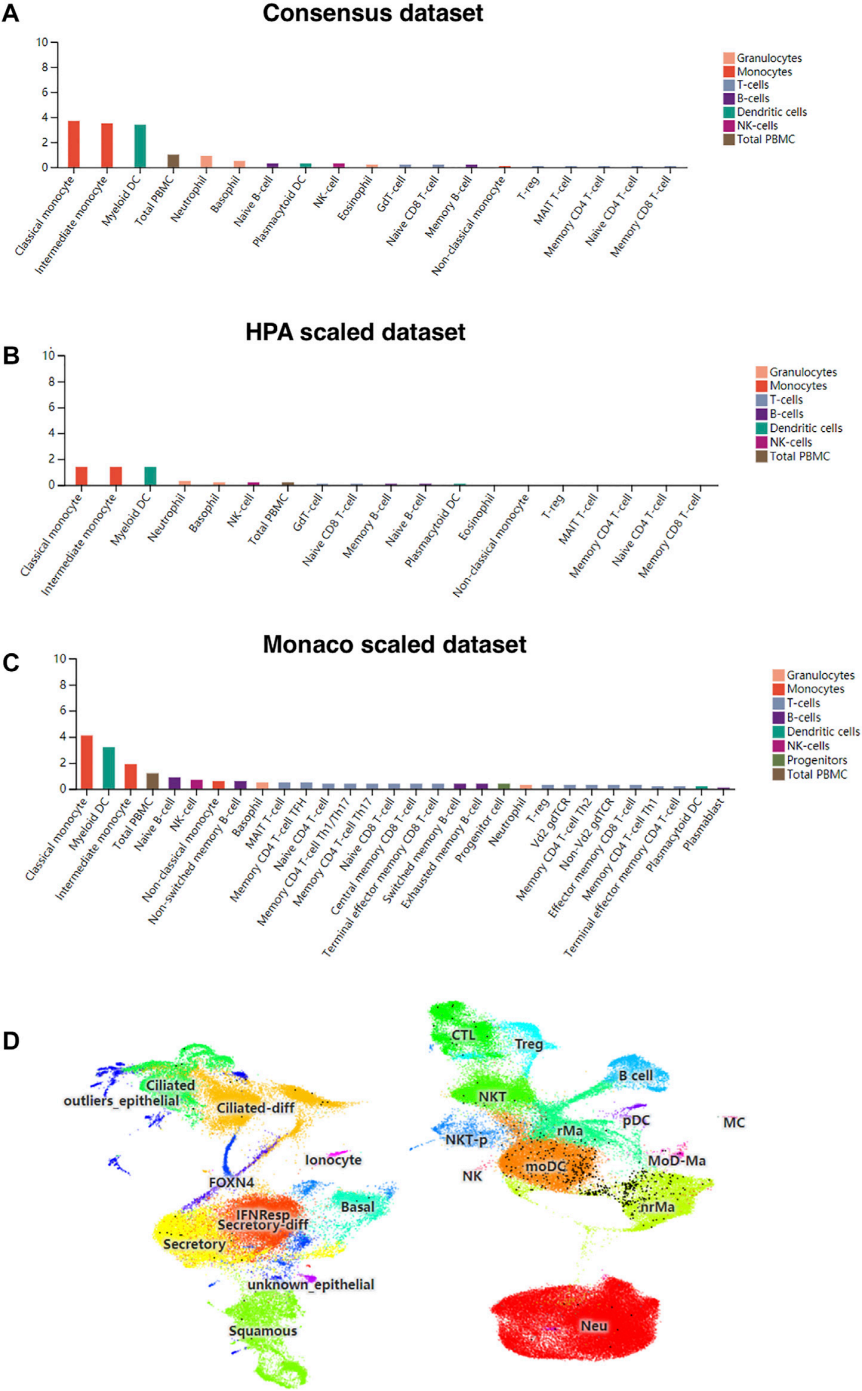
CD209 expression in human blood cells and nasopharyngeal cells in different datasets. **(A)** Consensus dataset, **(B)** HPA dataset, and **(C)** Monaco dataset. **(D)** Single-cell RNA-seq data for CD209 expression in nasopharynx immune cells of COVID-19 patients was downloaded from the UCSC cell browser portal (http://www.cells.ucsc.edu/). Each black dot represents the expression of CD209 in different cell types (total 135,600 cells). Abbreviation: MoDCs, monocyte-like inflammatory Dendritic Cells; nrMa, nonresident macrophages.

Patients severely infected by SARS-CoV-2 usually develop a “cytokine storm” (Mahallawi et al., 2018), which may be due to inflammation-induced lung injury and viral sepsis (Prompetchara et al., 2020). First, we investigated whether the liberation of these abnormal cytokines would be connected to the expression of CD209 across different types of immune cells in three different sub-datasets. Monocytes exhibited the highest CD209 expression, and DCs also expressed appreciable levels of CD209 (Figures 4A–C). Single-cell RNA-seq analysis also demonstrated similar results in monocyte-like inflammatory DCs (moDCs) and nonresident macrophages (nrMa) in the nasopharynx of COVID-19-infected patients (Figure 4D). Transcriptomic changes in COVID-19-related genes within virus-positive cells were highly upregulated in S100A8/A9, mainly in monocytes and megakaryocytes in peripheral blood, which might result in intensive “cytokine storms” that could be observed in severe patients (Ren et al., 2021). Myeloid dendritic cells, which have played a crucial role in adaptive immunity generation, are known for being able to present antigens toward T-cell surfaces and could sensitize naive T cells (Howard et al., 2004). These data illustrated that CD209 is widely expressed in various adaptive/innate immune cells, primarily in DC cells and monocytes, suggesting that CD209 is pivotal in the immune system and may account for the “cytokine storm” that occurs in some severe cases of SARS-CoV-2 infection.

### CD209 Expression Links Immune Cell Infiltration in Pan-Carcinoma Patients

**FIGURE 5:**
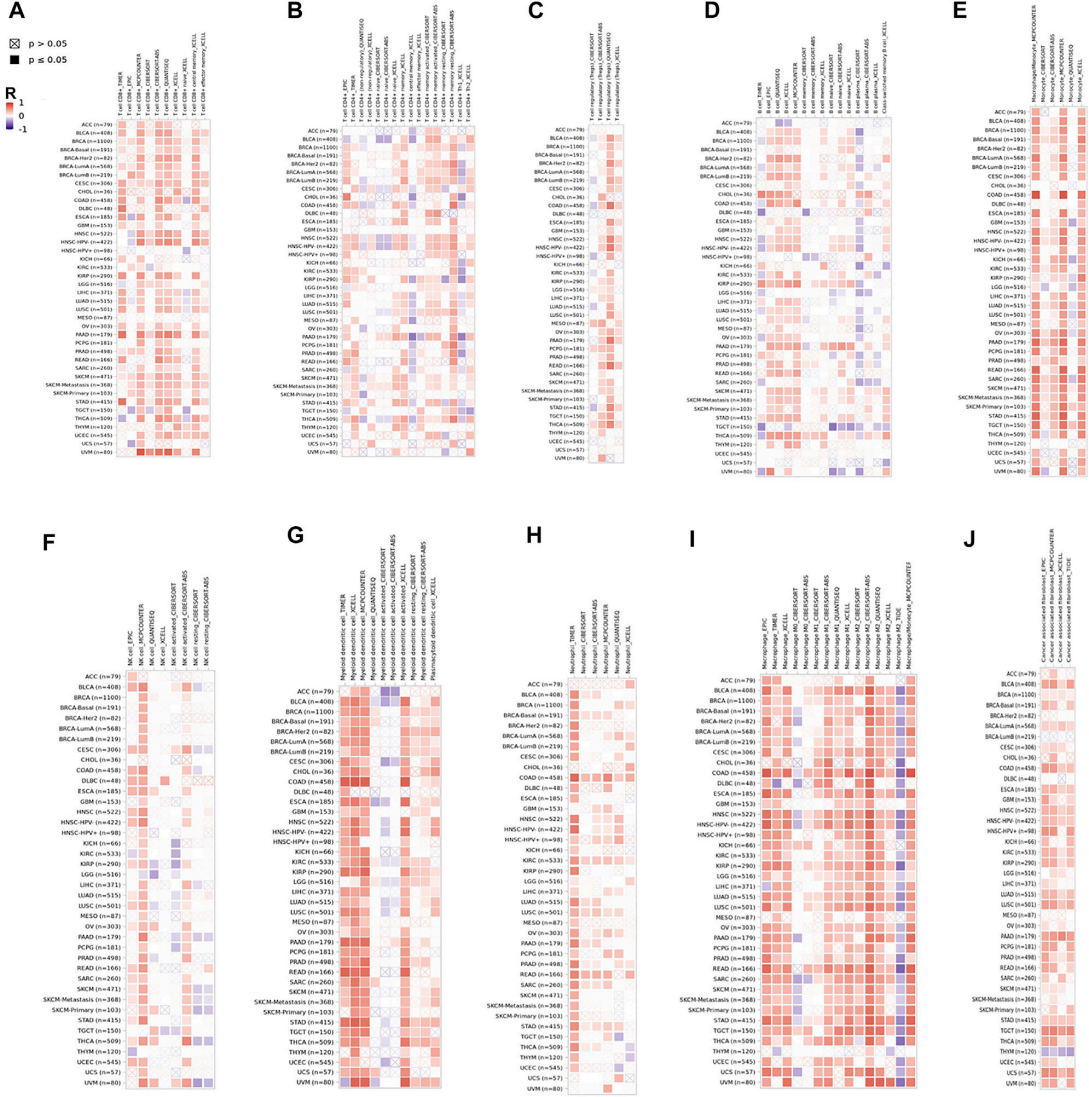
CD209 correlates with the infiltration of innate and adaptive immune cells in various cancer types. Correlation of CD209 with **(A)** the infiltration of CD8^+^ T cells, **(B)** CD4^+^ T cells, **(C)** Treg cells, (**D)** B cells, **(E)** monocytes cells, **(F)** NK cells, **(G)** dendritic cells, **(H)** neutrophils, **(I)** macrophages, and **(J)** cancer-associated fibroblasts in various cancer types analyzed by TIMER2 after purity adjustment. R: Spearman’s correlation coefficient. Spearman’s R (red): positive correlation, *p* < 0.05, r > 0; Spearman’s R (blue): negative correlation, *p* < 0.05, r < 0.

Tumor-infiltrating immune cells (TIICs) play a crucial role in tumorigenesis and progression in the tumor microenvironment (TME). Therefore, we first investigated whether CD209 expression was correlated with TIIC levels in various cancer types from TIMER. The results showed that the CD209 expression was evidently correlated with the infiltration of CD8^+^ T cells (Figure 5A), CD4^+^ T cells (Figure 5B), Treg cells (Figure 5C), B cells (Figure 5D), monocytes (Figure 5E), NK cells (Figure 5F), myeloid dendritic cells (Figure 5G), neutrophils (Figure 5H), macrophages (Figure 5I), and cancer-associated fibroblasts (Figure 5J) in pan-carcinoma patients.

**FIGURE 6:**
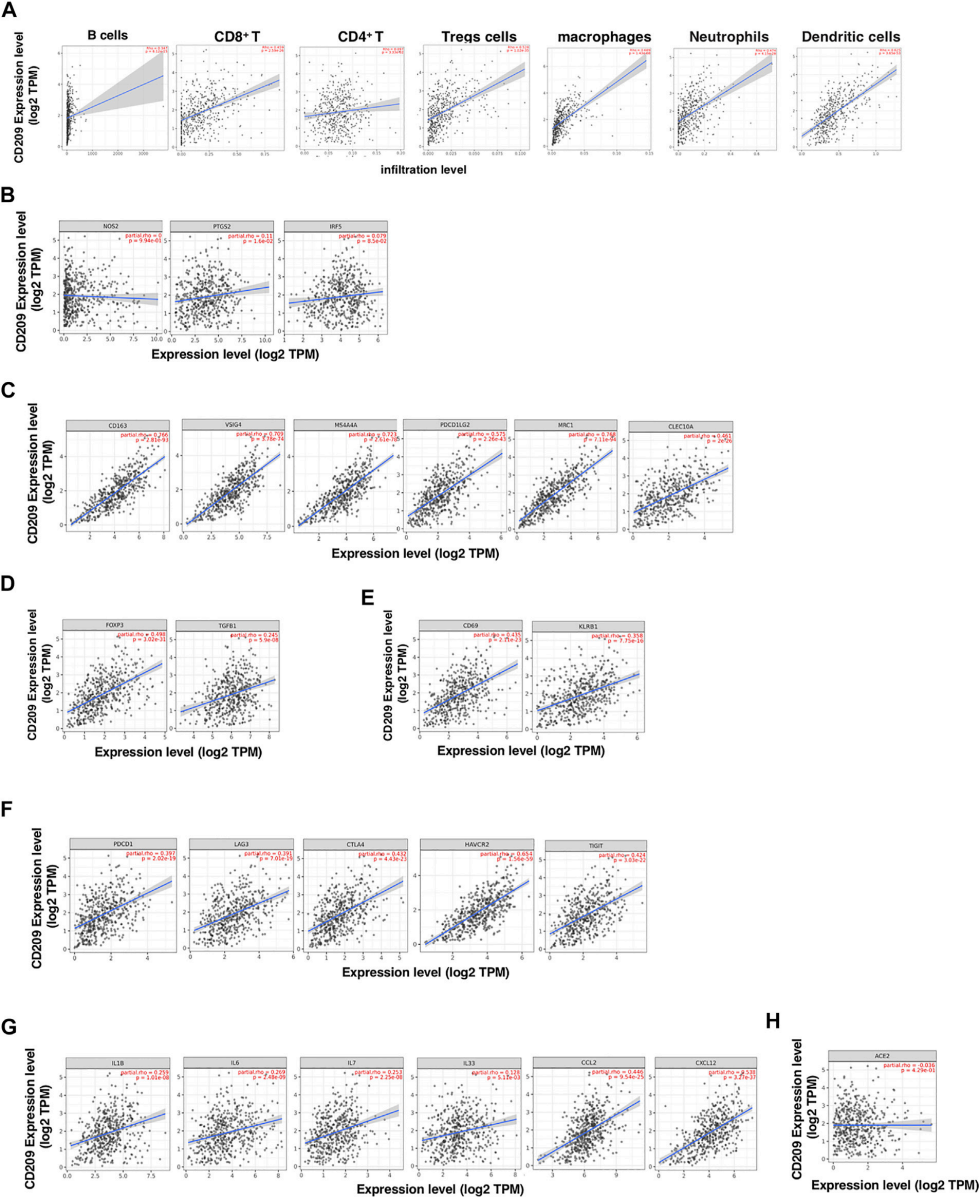
CD209 promotes the infiltration of immune cells and the release of cytokines in lung squamous cell carcinoma (LUSC) analyzed with TIMER. **(A)** Correlation of CD209 expression with the infiltration levels of different immune cells including B cells, CD8^+^ T, CD4^+^ T, Tregs, macrophages, neutrophils, and dendritic cells in LUSC analyzed with TIMER. **(B–E)** Scatter plots of correlations between CD209 expression with gene markers of **(B)** M1 macrophages, **(C)** M2 macrophages, **(D)** regulatory T cells, and **(E)** memory T cells in LUSC. **(F)** Scatter plots of correlations CD209 expression and T-cell exhaustion markers. **(G)** Scatter plots of correlations CD209 expression and inflammatory cytokines and chemokines. **(H)** Correlation of CD209 expression with ACE2 in LUSC. CD209 was used for the *y*-axis with gene symbols and on the *x*-axis, related marker genes are represented as gene symbols. The gene expression level was displayed using log2 TPM. Abbreviation: ACE2, angiotensinI-converting enzyme-2.

The higher expression of CD209, an alternative receptor of SARS-CoV-2, was associated with lower OS in patients with LUSC (Figure 3). Therefore, we further explored the correlation between CD209 and immune storms in LUSC patients. The CD209 expression showed significant positive correlations with tumor purity and infiltrating levels of immune cells in LUSC patients (Figure 6). In particular, the CD209 expression was found to be positively correlated with infiltrating levels of B cells (r = 0.229, *p* = 4.46e-06), macrophages (r = 0.645, *p* = 2.25e-57), CD4^+^ T cells (*p* = 3.28e-22, r = 0.424), neutrophils (r = 0.383, *p* = 4.29e-18), Treg cells (*p* = 9.21e-25, r = 0.447), CD8^+^ T cells (*p* = 4.25e-20, r = 0.403), and dendritic cells (*p* = 9.83e-45, r = 0.568) (Figure 6A). These findings strongly suggested that CD209 might play a specific role in tumor immune infiltration, especially the recruitment of macrophages and dendritic cells in LUSC.

To further study the correlation between CD209 and a variety of infiltrating immune cells, we explored the relationship between CD209 and various immune markers in LUSC, including M1/M2 macrophages (Figures 6B,C), Treg cells (Figure 6D), and memory T-cell markers (Figure 6E). Interestingly, we found that the CD209 expression level was positively associated with the immune markers of tumor-associated macrophages (TAMs) and M2 macrophages but not M1 macrophages (Figure 6B). There were significant correlations between CD209 and M2 marker genes, such as CD163 (r = 0.766, *p* = 2.81e-93), MS4A4A (r = 0.723, *p* = 2.61e-78), VSIG4 (r = 0.709, *p* = 3.78e-74), MRC1 (*p* = 2.11e-94, r = 0.768), CLEC10A (P = 2e-26, r = 0.461), and PDCD1LG2 (r = 0.573, *p* = 2.26e-43) (Figure 6C). Moreover, positive correlations were observed between CD209 and the infiltration levels of Treg cells with TGFB1 (*p* = 5.9e-08, r = 0.245) and FOXP3 (r = 0.498, *p* = 3.02e-31) (Figure 6D). In addition, we also found significant correlations between CD209 and marker genes of T-cell exhaustion, such as CTLA4 (*p* = 4.43e-23, r = 0.432), PD-1 (r = 0.397, *p* = 2.02e-19), TIGIT (*p* = 3.03e-22, r = 0.424), HAVCR2 (*p* = 1.56e-59, r = 0.654), and LAG3 (*p* = 7.01e-19, r = 0.391) (Figure 6F). We further found that CD209 expression significantly correlated with the levels of a panel of inflammatory cytokines and chemokines, including IL-1 (r = 0.259, *p* = 1.01e-08), IL-6 (r = 0.269, *p* = 2.48e-09), IL-7 (r = 0.253, *p* = 2.25e-08), IL-33 (r = 0.128, *p* = 9.72e-10), CCL2 (r = 0.446, *p* = 9.54e-25), and CXCL12 (r = 0.538, *p* = 3.27e-37) (Figure 6G). A previous study revealed that ACE2 showed a weak correlation with immune infiltration compared to CD209 (Shu et al., 2021). We further found that CD209 had a weak correlation with ACE2 in LUSC (*p* = 4.29e-1, cor = −0.036) (Figure 6H). In summary, these results revealed that CD209 was specifically correlated with immune infiltrating cells in specific tumors, which might ultimately contribute to the immune storm in SARS-CoV-2-infected cancer patients.

## Discussion

COVID-19 is a worldwide pandemic, while there is currently no effective treatment for this disease (Song et al., 2022). Cancer patients, smokers, and obese individuals are considered to be more susceptible to SARS-CoV-2 infection and have a poor prognosis due to the high expression of ACE2 (Liang et al., 2020; Al Heialy et al., 2020; Patanavanich and Glantz, 2020; Zhang et al., 2021), a primary receptor of SARS-CoV-2 (van Liempt et al., 2006; Op den Brouw et al., 2008; Jia et al., 2020). In addition to ACE2, other potential SARS-CoV-2 receptors for infection are indicated, including CD209 (Wang et al., 2021). Understanding the potential additional receptors of SARS-CoV-2 could be helpful to provide new insights and identify potential therapeutic targets for the better treatment of COVID-19 (Iyer et al., 2020; Rahimi, 2020; Smatti et al., 2020; Anisul et al., 2021; Dos Santos et al., 2021). CD209 (DC-SIGN) has been shown to be a receptor of various pathogens, such as yeast, HIV-1, and other specific viruses (Alam et al., 2021; Rahimi, 2020). It is worth noting that CD209 has been found to be a potential receptor for SARS and SARS-2 (Khoo et al., 2008; Di Maria et al., 2020).

In this study, we found that CD209 was expressed at high levels in the small intestine, adipose tissue, and lymph nodes (Figure 1), which implied that these sites may be mainly “attacked” by SARS-CoV-2 infection. The higher expression of CD209 in the small intestine might contribute to the fecal–oral transmission route of SARS-CoV-2 (Geurtsen et al., 2010). Previous studies have shown that ACE2 is barely detected in immune cells, such as T/B cells, which suggests that lymphocytopenia in patients with COVID-19 might be explained by the high expression of CD209 in lymphoid tissue (Zhang et al., 2021). Moreover, the elevated level of CD209 may be a possible reason for more severe clinical symptoms in COVID-19-infected obese patients.

Aging is a prognostic factor in SARC patients, and elevated CD209 was consistently detected in SARC patients with age (Figure 2). Furthermore, the CD209 expression is closely related to prognosis in some specific cancer types. Higher CD209 was associated with a poor prognosis in LUSC, KIRC, KIRP, and LHC patients (Figure 3). In this study, we demonstrated that CD209 (DC-SIGN) was abundantly expressed in many types of immune cells, especially in monocytes and myeloid DCs (Figure 4), which might contribute to the weakened immune response in elderly tumor patients.

Various immune cell types exhibit different expression levels of CD209, which might be attributed to the effects of algorithms in different databases. We also found that CD209 expression was correlated with diverse immune infiltration levels in various cancer types (Figure 5). Multiple studies have revealed that viruses and other pathogens targeting CD209 suppress the maturation of DCs, subsequently diminishing the production of cytokines and immune evasion (Kooyk et al., 2003; Zhang et al., 2020). In addition, the correlation between the CD209 expression and the marker genes of immune cells indicated the critical role of CD209 in the regulation of tumor immunology in LUSC patients (Figure 6). The gene markers of M1 macrophages, such as PTGS2 and NOS2, had weak correlations with CD209 expression, whereas M2 macrophage markers had moderate and strong correlations, revealing the potential regulatory role of CD209 in tumor-associated macrophage (TAM) polarization. Moreover, CD209 possesses the potential to activate memory T cells and Tregs and induce T-cell exhaustion. In view of the profiles of CD209 expression and their correlation with proinflammatory chemokines and cytokines and various immune cells in LUSC patients, CD209 may have great potential in exacerbating the “cytokine storm” in SARS-CoV-2-infected specific tumor patients (Figure 6).

**FIGURE 7:**
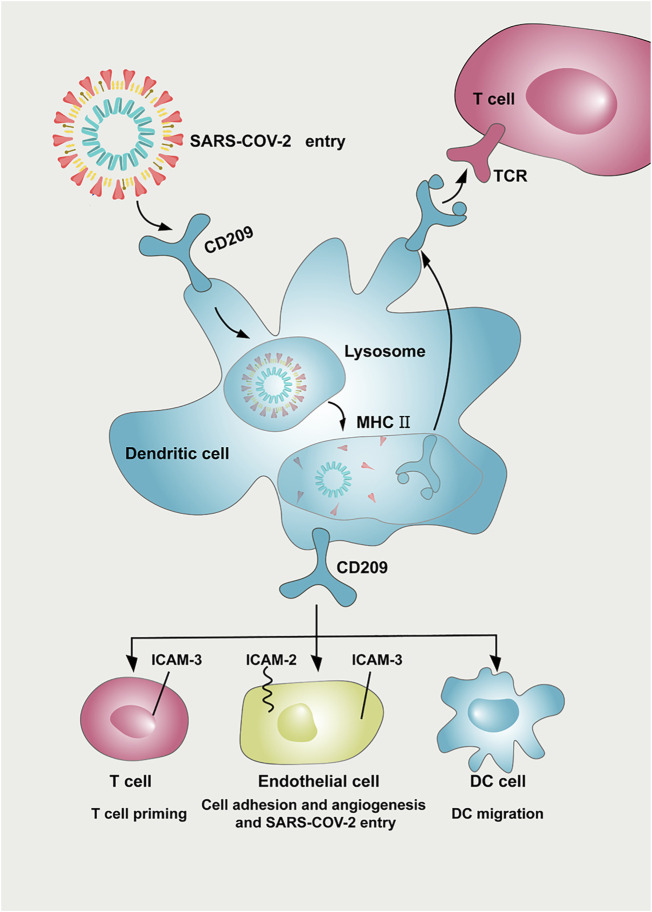
Potential implication of CD209 inhibitor in preventing SARS-CoV-2 damage in tumor patients. CD209 expressed in dendritic cells helps deliver SARS-CoV-2 antigen to the T-cell surface. CD209 mediates a variety of intercellular interactions among endothelial cells, DCs, and T cells. CD209 cell ligands (ICAM-2 and ICAM-3) are expressed in DCs, T cells, and endothelial cells, leading to multiple immunomodulation and other interactions between T cells, DCs, and endothelial cells. The CD209/ICAM-2/ICAM-3 interaction in endothelial cells can regulate endothelial adhesion and angiogenesis properties. As a potential receptor for SARS-CoV-2, CD209 inhibitors might also possess beneficial therapeutic effects on SARS-CoV-2 treatment and work together with other effective strategies. Abbreviation: ICAM-2, intercellular cell adhesion molecule-2; ICAM-3, intercellular cell adhesion molecule-3, DC: dendritic cell; MHC: major histocompatibility complex; TCR: T cell receptor.

In summary, CD209 is abundantly expressed in various immune cells, especially DCs and monocytes. The higher expression of CD209 triggers an immune response and may subsequently lead to a severe response to SARS-CoV-2 infection in cancer patients. Since CD209 is a potential specific receptor for SARS-CoV-2, these findings can provide a therapeutic clue for tumor patients and may prevent the risk of COVID-19 infection. CD209 antigen (D-mannose) might exhibit beneficial therapeutic effects on SARS-CoV-2 treatment together with other effective strategies in specific cancer patients (Figure 7). Further validation of animal experiments and clinical trials is required to evaluate the effect of anti-CD209 therapy in specific cancer patients with SARS-CoV-2 infection.

## Data Availability

The original contributions presented in the study are included in the article/Supplementary Material; further inquiries can be directed to the corresponding authors.
